# Severe Rhabdomyolysis With Acute Kidney Injury Triggered by Influenza A and Strenuous Exercise in a Healthy Adolescent: A Dual-Hit Mechanism

**DOI:** 10.7759/cureus.104029

**Published:** 2026-02-21

**Authors:** Iliass Benaini, Mohamed Zakaria Bouayed, Younes Oujidi, Mahmoud Faouzi Ismaili, Ilyass Laaribi, Ikram Zaid, Houssam Bkiyar

**Affiliations:** 1 Intensive Care and Anesthesia Department, Mohammed VI University Hospital, Oujda, MAR; 2 Simulation Center, Laboratory of Anatomy, Microsurgery, Experimental Surgery and Medical Simulation (LAMESMS), Faculty of Medicine and Pharmacy, Mohammed I University, Oujda, MAR

**Keywords:** acute kidney injury, exercise, influenza a, renal replacement therapy, rhabdomyolysis

## Abstract

Rhabdomyolysis is a serious clinical condition often associated with serious complications and can be life-threatening in nature. The following is a case report of a healthy 17-year-old male adolescent who presented with rhabdomyolysis due to a dual hit of Influenza A and excessive physical exertion. Acute onset of myalgia, weakness in the lower extremities, and dark urine warranted his hospitalization in the intensive care unit for critical monitoring and investigation. The laboratory findings indicated a high creatine kinase level of more than 60,000 U/L, severe metabolic acidosis, hyperkalemia, and renal failure. Despite aggressive fluid resuscitation, persistent anuria and metabolic derangements warranted renal replacement therapy with continuous veno-venous hemofiltration followed by intermittent hemodialysis, resulting in progressive recovery of renal function and clinical improvement. This case highlights the importance of recognizing rhabdomyolysis in patients presenting with acute neuromuscular symptoms after febrile illness and strenuous exercise, emphasizing the need for early diagnosis and prompt supportive management to prevent irreversible renal injury.

## Introduction

Rhabdomyolysis is a potentially life-threatening entity caused by the rapid destruction of skeletal muscle fibers, with leakage of cellular contents such as creatine kinase (CK), myoglobin, and electrolytes into the bloodstream. The manifestations range from mild muscle pain to severe metabolic abnormalities, acute kidney failure (AKI), and systemic organ failure [1,2].

Several etiologies, such as trauma, drugs, toxins, infectious diseases, metabolic disorders, and severe physical exertion, have been incriminated. Some infectious etiologies, such as Influenza A, are among the more common infectious causes [3].

Exertional forms of rhabdomyolysis have been reported, and this type of rhabdomyolysis occurs more commonly after unusual and intense exercise, along with dehydration and febrile illness [4]. However, there is scarce literature on severe rhabdomyolysis resulting from the combined effect of a viral infection and intense exercise. We report a case of severe rhabdomyolysis complicated by stage 3 AKI requiring renal replacement therapy, resulting from Influenza A infection and intense physical exercise in a previously healthy adolescent.

## Case presentation

A 17-year-old previously healthy male presented with progressive asthenia, severe myalgia in the thighs and calves, and inability to stand and walk. Two days prior to presentation, he experienced a flu-like syndrome of malaise and myalgia with subjective fever without any objective measurements taken. He went on to participate in strenuous physical activity in the form of three consecutive football matches over a period of five hours, followed by short intervals of rest in the setting of poor oral intake and dehydration. Over the course of 24 hours, he went on to worsen in condition with increased muscle pain, generalized myalgia measuring 7/10 in the visual analog scale [5], weakness of the lower limb, and dark urine.

Initial diagnostic hypothesis included Guillain-Barré syndrome, acute inflammatory myositis or autoimmune myopathy, infectious or exertional rhabdomyolysis, and metabolic or electrolyte disorders affecting neuromuscular function. The rapidly progressive lower-limb weakness, functional impairment, and risk of respiratory compromise raised concern for potentially life-threatening neuromuscular or metabolic conditions, thereby warranting admission to the intensive care unit for close monitoring.

Upon admission, the patient was afebrile (36.8 °C), oriented, and fully conscious with a Glasgow Coma Scale [6] of 15; he was hemodynamically stable, with a non-invasive blood pressure of 125/55 mmHg and a heart rate of 60 bpm. Respiratory parameters were normal, with a respiratory rate of 18 breaths per minute and oxygen pulse saturation at 98% on ambient air. The diuresis was markedly reduced at 0.10 mL/kg/h, with dark-colored urine. The neurological examination showed a reduced strength of the lower limb muscle tone, graded 2/5 on the right and 3/5 on the left side, with slightly decreased sensitivity.

Laboratory studies, as outlined in Table 1, revealed severe rhabdomyolysis with markedly increased creatine phosphokinase (CPK) levels above 60,000 U/L and AKI, classified as stage 3 according to the Kidney Disease: Improving Global Outcomes (KDIGO) criteria, hyperkalemia, metabolic acidosis, and hyperphosphatemia. The high aspartate aminotransferase/alanine aminotransferase (AST/ALT) ratio indicated rhabdomyolysis with increased muscle enzymes. RT-PCR studies confirmed the diagnosis of Influenza A virus infection. Serologies for hepatitis A (IgM), hepatitis B surface antigen/core IgM, hepatitis C, human immunodeficiency virus (HIV), Cytomegalovirus, and Epstein-Barr Virus were negative. Arterial blood gas studies confirmed severe metabolic acidosis (Table 1).

**Table 1:** Overview of laboratory findings on admission

Biological data	On Admission	Normal range
Hemoglobin (g/dL)	13.1	12.5–17.5
White blood cells (×10⁹/L)	17.4	4.0–10.0
Platelets (×10⁹/L)	309	150–400
Creatinine (mg/dL)	12.9	0.7–1.2
Urea (g/L)	2.12	0.18–0.45
Creatine phosphokinase (U/L)	62,589	46–171
Aspartate aminotransferase (U/L)	5334	10–35
Alanine aminotransferase (U/L)	1404	8–35
Lactate dehydrogenase (U/L)	3325	125–220
Sodium (mmol/L)	127	135–145
Potassium (mmol/L)	6.1	3.5–5.2
Chloride (mmol/L)	99	98–107
Calcium (mg/L)	54	85–105
Phosphate (mg/L)	67.6	25–45
Albumin (g/L)	37	35–52
pH	7.21	7.35–7.45
PaCO₂ (mmHg)	22.5	35–45
PaO₂ (mmHg)	90	75–100
HCO₃⁻ (mmol/L)	14	22–26
Lactate (mmol/L)	0.65	<2

Based on the diagnostic considerations, we conducted further tests. Lumbar puncture was normal, while results from electromyoneurography did not point to any abnormalities, hence ruling out acute polyradiculoneuritis. Imaging studies that included cerebral computed tomography, renal-bladder-prostatic ultrasound, and Doppler ultrasound of lower limb vessels also did not indicate any abnormalities. A muscle biopsy on the right deltoid region to rule out inflammatory and autoimmune myopathies was normal.

The diagnosis of severe rhabdomyolysis secondary to a dual-hit mechanism, Influenza A infection and intense exertion, was retained.

Aggressive IV hydration with Ringer's lactate solution at a rate of 1500 mL/h and close monitoring for signs of fluid overload were undertaken. Hyperkalemia treatment involved the use of salbutamol, sodium polystyrene sulfonate, and an insulin and glucose regimen. On day 2 of hospitalization, continuous veno-venous hemofiltration (CVVH) was initiated for anuria for more than 24 hours, significant metabolic acidosis, and hyperkalemia despite optimal treatment. The CVVH treatment session was undertaken through a 16 cm right internal jugular catheter with blood flow at 150 mL/min, dialysate flow at 2700 mL/h, a temperature of 37°C, no ultrafiltration, and anticoagulation using heparin. The treatment session lasted for 12 hours and resulted in normalization of potassium levels to 4.5 mmol/L, pH to 7.40, and reduction of blood urea and creatinine levels to 1.70 g/L and 9.25 mg/dL, respectively. However, diuresis did not ensue, justifying intermittent hemodialysis sessions on days 3, 4, 6, and 7. Biochemical and clinical parameters showed evidence of improvement, including reduction of blood creatinine and urea levels to 4.82 mg/dL and 1.18 g/L, respectively. In addition, CPK levels were also significantly reduced to 10,480 U/L. Lactate dehydrogenase (LDH) and liver enzymes were normalized. The anuria persisted for some days, but diuresis gradually ensued on day 5 of hospitalization. The musculoskeletal symptoms also gradually improved. The patient was transferred to nephrology on day 8 and discharged on day 11 with normalized renal function and progressive functional recovery.

**Figure 1:**
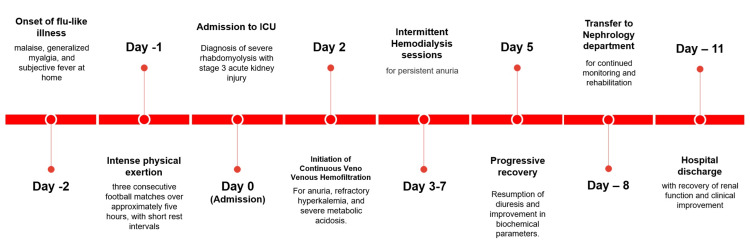
Clinical timeline of disease progression and management

The chronological sequence of clinical events is summarized in Figure 1.

## Discussion

This case illustrates a severe form of rhabdomyolysis resulting from a probable synergistic interaction between Influenza A infection and intense physical exertion in the setting of dehydration.

Rhabdomyolysis is a multifaceted medical entity characterized by the rapid breakdown of injured skeletal muscle tissue, resulting in the release of intracellular components, including myoglobin, CK, aldolase, LDH, and various electrolytes into the circulation and interstitial spaces. Its true incidence is difficult to assess [7], although epidemiological data suggest an increased prevalence among African-Americans, patients younger than 10 years old or older than 60 years old, and patients with morbid obesity [8]. The clinical spectrum of rhabdomyolysis ranges from asymptomatic CPK elevation to severe, life-threatening cases associated with electrolyte imbalances such as hyperkalemia, metabolic acidosis, hyperphosphatemia, and/or dyscalcemia, as well as other complications like AKI, compartmental syndrome, and disseminated intravascular coagulation [1,2,9].

Genetic or metabolic predispositions may play a role in the occurrence of rhabdomyolysis [10]. Conditions such as carnitine palmitoyl-transferase II (CPT II) deficiency and Ryanodine receptor 1 (RYR1) related susceptibility have been associated with the occurrence of recurrent or severe episodes of rhabdomyolysis precipitated by infections, physical activities, or other conditions [10].

Various infectious agents have been associated with rhabdomyolysis. Among these, viral infections are particularly frequent. Influenza viruses, especially Influenza A, are the most commonly incriminated, accounting for a significant proportion of viral-induced rhabdomyolysis cases [11,12], During the 2009 H1N1 pandemic, caused by the virulent A/California/07/2009 strain, several studies reported a high incidence of elevated CK levels correlated with disease severity, renal dysfunction, and prolonged mechanical ventilation [13-15].

The precise pathogenesis of rhabdomyolysis associated with Influenza A infection remains debated. Proposed hypotheses include direct viral invasion of myocytes, immune-mediated muscle injury resulting from cytokine release, and toxic viral metabolites or circulating inflammatory mediators that compromise muscle integrity [12].

In parallel, exertional rhabdomyolysis typically occurs after unaccustomed or prolonged physical activity, particularly when associated with dehydration, febrile illness, high ambient temperature or humidity, genetic susceptibility, and use of performance-enhancing drugs [4,10,16]. Football involves repeated eccentric muscle contractions and high-intensity intermittent exertion, which may predispose to muscle injury. This form of rhabdomyolysis is reported more frequently in men, partly due to more intense exercise patterns [17]. The pathophysiological mechanism of exercise-induced rhabdomyolysis involves depletion of adenosine triphosphate during sustained muscle activity, leading to failure of calcium homeostasis, activation of proteases and phospholipase A₂, and subsequent cell membrane rupture. Acute renal failure results from myoglobin-induced tubular toxicity and renal vasoconstriction secondary to hypovolemia [18].

Apart from the co-occurrence of Influenza A infection and strenuous physical activity that was observed in the current patient, there is emerging evidence that a potential synergistic pathophysiology may exist in which infection-induced immune activation results in a predisposition to muscle injury [19]. The presence of a viral infection may result in the release of pro-inflammatory cytokines and chemokines that enhance inflammation and oxidative stress and compromise the integrity of the sarcolemma, which may result in a predisposition to muscle fiber injury during strenuous physical activity [20].

In this patient, the combination of influenza-related myositis, dehydration, and repeated intense activity likely created a “dual-hit” mechanism leading to massive muscle breakdown and severe AKI. Although each of these factors independently represents a recognized cause of rhabdomyolysis, their simultaneous occurrence may markedly increase the severity of muscle injury.

Early aggressive fluid resuscitation remains the cornerstone of management, aiming to dilute circulating myoglobin and prevent pigment-induced nephropathy. When conservative therapy fails, renal replacement therapy is warranted and described [1,2,18], in order to correct hyperkalemia, metabolic acidosis, and uremia, as illustrated in this case.

This case also highlights the importance of clinical reasoning in the diagnostic approach to acute neuromuscular weakness. Guillain-Barré syndrome, inflammatory myopathy, and metabolic causes must be considered and excluded through appropriate investigations. Early identification of rhabdomyolysis allows prompt initiation of treatment and may significantly improve outcomes.

It is noteworthy that in the acute phase of rhabdomyolysis, the result of the muscle biopsy could be non-specific, and in certain instances, follow-up investigation after clinical recovery could be considered in the differential diagnosis of metabolic myopathies [21]. In the current patient, however, the presence of complete clinical and biochemical recovery without any evidence of relapse did not warrant further invasive investigation, including advanced genetic screening.

The favorable recovery observed in this patient underscores the importance of early recognition, aggressive supportive therapy, and multidisciplinary management.

From a practical perspective, febrile illness should be regarded as a temporary contraindication to high-intensity physical exertion, even in young athletes, as systemic inflammation may predispose to severe muscle injury.

## Conclusions

In summary, this case highlights the complex interplay between infectious and exertional factors in the pathogenesis of rhabdomyolysis. The dual trigger of Influenza A infection and intense exercise underscores the need for clinical vigilance in patients presenting with muscle pain, weakness, or dark urine during or after febrile illness. Early diagnosis and timely initiation of aggressive hydration and renal replacement therapy remain critical to prevent irreversible renal damage and improve survival. Furthermore, given that severe (KDIGO stage 3) AKI in adolescence may increase the risk of subsequent chronic kidney disease, structured long-term nephrological follow-up is essential in these patients.
